# Acute stress impairs attentional control and emotional processing in social anxiety

**DOI:** 10.1016/j.isci.2026.115767

**Published:** 2026-04-17

**Authors:** Mozhao Li, Alex H.K. Wong, Bernadette von Dawans, Gregor Domes, Matthias J. Wieser

**Affiliations:** 1 Department of Psychology, Education, and Child Studies, Erasmus University Rotterdam, 3062 PA Rotterdam, the Netherlands; 2 Biological and Clinical Psychology, Department of Psychology, University of Trier, 54296 Trier, Germany; 3 Institute for Cognitive and Affective Neuroscience, University of Trier, 54296 Trier, Germany

**Keywords:** Health sciences, Medicine, Psychiatry, Psychology

## Abstract

Processing emotional faces poses a particular challenge for socially anxious individuals. Acute social stress may further alter attentional allocation and emotional processing, yet the temporal neural dynamics of these changes remain unclear. Using an electroencephalogram (EEG), we examined attentional bias and face processing through an attentional threat bias task and a passive face-viewing task following stress induction. Results showed that acute stress biased a sustained visuospatial attention toward the left visual field, which indicates reduced flexibility in reallocating attention across competing affective stimuli. Stress also broadly reduced event-related potential (ERP) amplitudes and attenuated frontal alpha event-related desynchronization (alpha ERD), suggesting diminished attentional resource availability and cortical control. Notably, early rapid responses to threatening faces remain intact. Together, these findings indicate that acute stress reorganizes attentional processing by depleting resources required for sustained attention and regulatory engagement, potentially exacerbating social information processing vulnerabilities in social anxiety.

## Highlights

• Acute stress biases sustained visuospatial attention toward the left visual field
• Acute stress depletes attentional resources and reduces overall attentional engagement
• Acute stress preserves early threat vigilance but reduces later processing

## Graphical abstract

**Figure:**
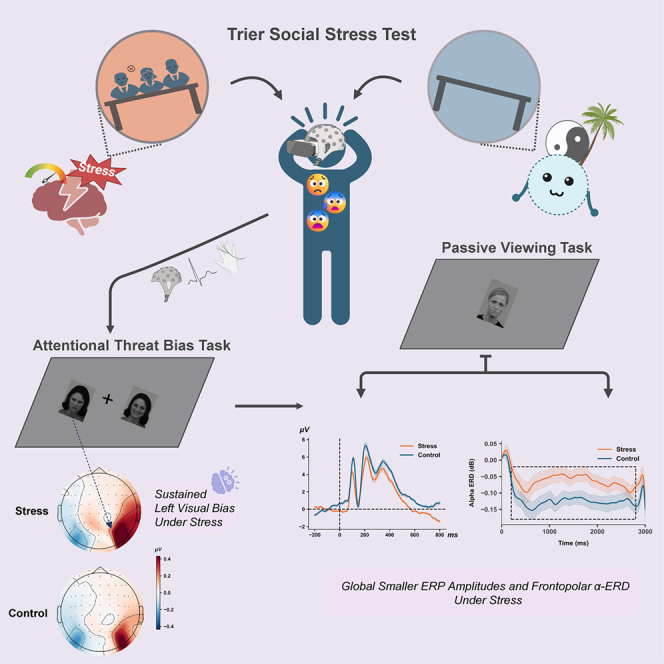

## Introduction

Social anxiety disorder (SAD) is characterized by excessive fear of negative social evaluation and a maladaptive focus on threat-related information.^1^^,^^2^^,^^3^^,^^4^ These biases underlie core symptoms of SAD, including hypervigilance to social threat, exaggerated the interpretation of ambiguous cues, and difficulty disengaging from aversive stimuli.^5^^,^^6^^,^^7^ As faces are the primary carriers of interpersonal information, they frequently serve as salient threat cues for socially anxious individuals.^8^^,^^9^^,^^10^ Empirical evidence shows that individuals with SAD consistently exhibit enhanced face- and emotion-specific event-related potential (ERP) responses, regardless of valence,^11^ which reflects exaggerated structural encoding and perceptual sensitivity.^11^^,^^12^ They are particularly sensitive to anger, displaying faster detection,^13^ greater attention,^14^^,^^15^ more negative interpretations,^16^ and difficulty disengaging from angry or even neutral faces.^17^ In some cases, positive stimuli can be treated similarly to threat, because of the anticipation of future competition or group-level social dynamics.^18^^,^^19^^,^^20^ Collectively, these patterns describe attentional threat bias (ATB)—the preferential allocation of attention to potential threats.^21^

These biases can be further shaped by acute stress responses.^22^ Rapid activation of the sympathetic-adrenal-medullary (SAM) axis recruits the corticotropin-releasing factor-locus coeruleus-norepinephrine circuit, a key modulator of forebrain activity.^23^ This catecholaminergic surge heightens amygdala reactivity and suppresses prefrontal cortex (PFC) regulation.^24^^,^^25^^,^^26^ In anxiety disorders, such an imbalance is believed to amplify automatic attentional biases toward threats and delayed disengagement from them.^7^ The sympathetic predominance supports defensive readiness,^27^ but comes at the cost of heightened distractibility,^28^ reduced task performance,^29^ as well as impaired selective attention^30^ and working memory.^31^ In contrast, the slower hypothalamic-pituitary-adrenocortical (HPA) axis, through delayed cortisol release, aids longer-term cognitive restoration and re-establishment of regulatory balance.^32^

If stress indeed shifts the balance toward heightened salience detection and reduced PFC control, such changes should manifest in time-resolved neural signatures. Electrophysiological studies indicate that stress can augment both early perceptual and later evaluative stages of emotional processing.^33^^,^^34^ Extensive studies have reported increased ERP amplitudes to negative or threatening faces, such as higher N170 and EPN to angry faces under a social stressor,^35^ larger N2pc and earlier LPP onset following stress,^36^ and generally enhanced P1 along with selective P2 increases to angry faces even under prolonged academic stress.^37^

Yet enhanced emotion-related neural responses do not necessarily translate into consistent attentional processing. Some studies have reported enhanced alertness under acute stress,^38^ whereas others have found disrupted selective attention and greater distractibility,^28^^,^^39^ and still others have observed no difference.^40^ The inverted-U model of catecholamine/dopamine modulation offers one account: Mild stress may facilitate cognitive control, but excessive stress generally impairs it,^41^^,^^42^ yet varying stress intensities and sample characteristics complicate direct comparisons. Interestingly, even when stress reduces accuracy in detecting threatening faces within a crowd, the ability to rapidly identify them—or the attentional advantage—often persists.^43^ These inconsistencies highlight the need for broader neural markers that capture the full window of processing.

At the spectral level, alpha event-related desynchronization (alpha ERD) indexes task-related cortical excitation and top-down filtering via frontoparietal networks.^44^^,^^45^^,^^46^^,^^47^^,^^48^^,^^49^ Acute stress can disrupt these dynamics and related large-scale network interactions.^50^ The lateral and orbital regions of the frontal pole, which are engaged in higher-order functions such as attention, working memory, and emotion regulation^51^^,^^52^ typically reduce activity under stress.^53^ Additionally, impaired medial frontal-thalamic pathways can hinder the default mode network (DMN) deactivation and redirect processing inward.^54^ Alpha ERD thus provides a useful marker of the brain’s ability to mobilize attentional performance and top-down control under stress.

Although these mechanistic insights are well characterized, far fewer studies have tested how acute stress shapes socio-emotional functioning in social anxiety. Behaviorally, socially anxious individuals display greater avoidance of social signals,^55^ show reduced positive responsiveness to social rejection,^56^ and engage in less prosocial behavior^57^ or show poorer conversational performance.^58^ Beyond behavior, stress-induced cognitive changes have also been reported in this population, including poorer working memory,^59^ altered attentional engagement with a bias to disgust that relates to post-event processing,^60^ and improved inhibition control in individuals with high social anxiety.^61^ These findings suggest that acute stress does not uniformly impair cognition in social anxiety but may reorganize processing priorities toward social threat.

However, despite evidence of altered functioning under stress, it remains unclear how acute stress modulates the temporal dynamics of attention and emotional processing in the socially anxious population. Classic paradigms such as the dot-probe, emotional Stroop, and visual search tasks capture only discrete snapshots of attentional direction and rely heavily on explicit response demands.^62^^,^^63^^,^^64^^,^^65^ Consequently, they cannot track continuous transitions across distinct stages (*e.g*., orienting vs. disengagement) and are often confounded by task-irrelevant processes that obscure underlying attentional mechanisms.^66^^,^^67^^,^^68^^,^^69^^,^^70^

**Figure 1:**
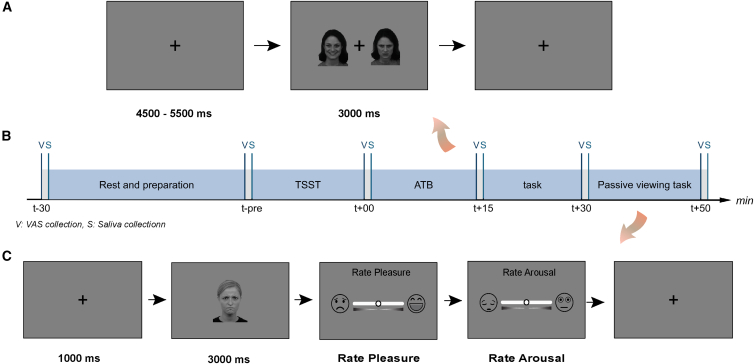
Schematic illustration of the experimental paradigms (A) Attentional threat bias (ATB) task. (B) Timeline of the experiment protocol. Time points are relative to the Trier Social Stress Test (TSST). (C) Passive viewing task.

To address these methodological limitations, we adopted a multidimensional approach that combined steady-state visual evoked potentials (ssVEPs), ERPs, and frontal pole alpha ERD. ssVEPs allow continuous, frequency-tagged tracking of covert attention.^14^^,^^71^^,^^72^ ERPs indicate discrete processing stages with high temporal precision, and alpha ERD offers complementary insights into the attentional engagement and regulatory capacity. Acute stress was induced using a virtual reality adaptation of the Trier Social Stress Test (TSST; Open TSST-VR, translated and adapted into Dutch),^73^ which elicits psychosocial stress responses while increasing ecological validity for socially anxious participants.^74^^,^^75^ Participants then completed two electroencephalogram (EEG) tasks: an ATB task, designed to assess attention allocation toward emotional faces, and a passive face-viewing task, in which participants rated the valence and arousal of facial expressions (Figure 1). Behavioral, electrophysiological, and psychological measures, including electrodermal activity and heart rate (HR),^76^^,^^77^^,^^78^ were collected to capture multi-level responses.

We hypothesized that in socially anxious individuals, acute stress would (1) bias attention toward angry faces in the ATB task and (2) amplify neural and psychophysiological responses to angry faces during passive viewing. By integrating continuous and time-resolved measures, this study aims to provide a comprehensive understanding of acute stress effects on attention and emotional processing in the context of social anxiety symptoms, thereby informing more precise interventions that promote not only symptom relief but also resilience to everyday stressors.

## Results

### Stress assessment

#### Subjective stress response

**Figure 2:**
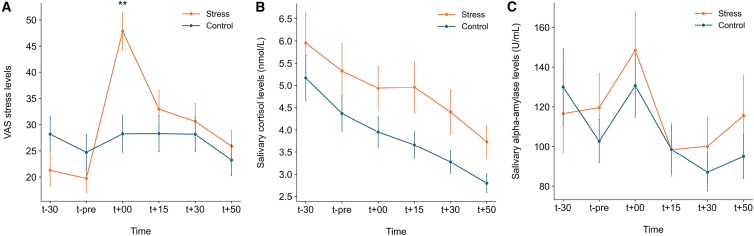
Stress responses to the Trier Social Stress Test (TSST) (A) Subjective stress ratings (VAS) across time. The stress group reported significantly higher stress after the TSST, with no group differences at other time points (group × time interaction, *F*(3.61, 281.67) = 7.69, *p* < 0.001, $\eta_{p}^{2}=$ 0.090; *n* = 40 participants per group). (B) Salivary cortisol levels across time. Cortisol levels decreased over time in both groups, with no group differences (main effect of time, *F*(1.67, 130.47) = 17.66, *p* < 0.001, $\eta_{p}^{2}=$ 0.19; *n* = 40 participants per group). (C) Salivary alpha-amylase (sAA) levels across time. sAA levels increased immediately after the TSST in both groups, with no group differences (main effect of time, *F*(3.14, 219.94) = 13.01, *p* < 0.001, $\eta_{p}^{2}=$ 0.16; *n* = 36 participants per group). Time points are relative to the TSST. Data and error bars show mean ± standard error (SE). Statistical significance was assessed using two-way ANOVA (group × time point) with Bonferroni correction. ∗∗ indicates *p* < 0.01 for between-group comparisons at the corresponding time point.

The main effect of time was significant, *F*(3.61, 281.67) = 11.51, *p* < 0.001, $\eta_{p}^{2}=$ 0.13. A significant group × time interaction effect was also observed, *F*(3.61, 281.67) = 7.69, *p* < 0.001, $\eta_{p}^{2}=$ 0.090, with the stress group reported significantly higher stress levels than the control group immediately after the TSST (t+00; *p* < 0.001; Figure 2A). No significant group differences were found at any other timepoints, nor was there a main effect of group (all *ps* > 0.16).

#### Physiological stress response

For cortisol levels, the analysis revealed a significant decrease over time, *F*(1.67, 130.47) = 17.66, *p* < 0.001, $\eta_{p}^{2}=$ 0.19 (Figure 2B). However, this continuous decline across multiple timepoints, regardless of group, likely reflects a general temporal drop rather than a stress-specific effect. For sAA levels, a significant time effect was also found (*F*(3.14, 219.94) = 13.01, *p* < 0.001, $\eta_{p}^{2}=$ 0.16), with a significant rise immediately after stress offset for both groups (t+00; *ps* < 0.001) (Figure 2C). No significant group or interaction effects were observed in either cortisol or sAA concentrations, as well as when using the AUCg or AUCi measures (*ps* > 0.085).

### Behavioral results

#### Affective ratings

Table 1 presents the mean ratings for arousal and valence by group. For arousal, the main effect of expression was significant, *F*(1.75, 136.32) = 33.96, *p* < 0.001, $\eta_{p}^{2}=$ 0.30. Angry and happy expressions were rated as significantly more arousing than neutral faces (*ps* < 0.001). No group or interaction effect was found (*ps* > 0.16). For valence, the main effect of expression was also significant, *F*(1.26, 98.61) = 703.11, *p* < 0.001, $\eta_{p}^{2}=$ 0.90. Additionally, a significant expression × group interaction was found, *F*(1.26, 98.61) = 4.19, *p* = 0.034, $\eta_{p}^{2}=$ 0.051, with the stress group rating the angry expressions as significantly more unpleasant than controls (*p* = 0.022), whereas no significant differences were observed for happy or neutral expressions. No group effect was found (*p* = 0.99).

**Table 1:** Arousal and valence ratings by group and expression

		Stress (M ± SD)	Control (M ± SD)
Arousal	Angry	0.58 ± 0.14	0.55 ± 0.14
	Happy	0.52 ± 0.17	0.51 ± 0.13
	Neutral	0.37 ± 0.14	0.42 ± 0.11
Valence	Angry	0.21 ± 0.086	0.26 ± 0.095
	Happy	0.79 ± 0.080	0.75 ± 0.10
	Neutral	0.43 ± 0.057	0.43 ± 0.050

Note: M = mean; SD = standard deviation; *n* = 40 participants per group.

### Physiological data

#### SCR

In the ATB task, 30 participants in the stress group and 28 participants in the control group produced valid SCRs and were included in the analysis. The main effect of emotion combination was significant, *F*(1.55, 86.71) = 4.85, *p* = 0.016, $\eta_{p}^{2}=$ 0.080, with happy-neutral combinations eliciting significantly larger SCRs than angry-neutral combinations (*p* < 0.001). No other effects reached significance (*ps* > 0.37). In the passive viewing task, 23 participants in the stress group and 20 participants in the control group were included. No significant main effects or interaction effects were found (*ps* > 0.24).

#### HR

No significant effects were found for HR in any time window of the ATB task (*ps* > 0.051) or in the 0–3000 ms window of the passive viewing task (*ps* > 0.42).

### EEG results

#### ssVEP amplitudes of the ATB task

**Figure 3:**
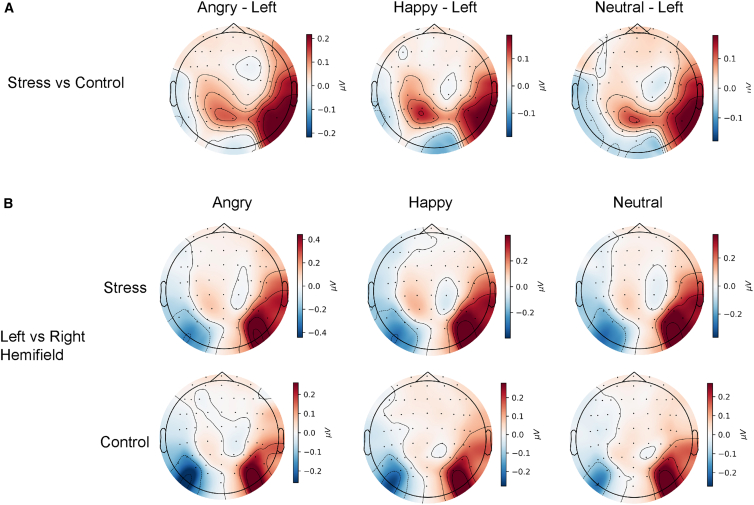
Topographic maps of ssVEP amplitude differences in the attentional threat bias (ATB) task (A) Group difference topographies for each expression combination and hemifield across the entire time window (*n* = 40 participants per group). Positive values indicate higher amplitudes in the stress group, whereas negative values indicate higher amplitudes in the control group. The stress group showed higher ssVEP amplitudes over occipital and parieto-occipital regions in the right hemisphere, corresponding to greater attentional bias toward the left hemifield across expression combinations compared to the control group. (B) Hemispheric lateralization effects for each expression combination across the entire time window (*n* = 40 participants per group). Positive values indicate greater right-hemisphere activity, whereas negative values indicate greater left-hemisphere activity. The stress group showed increased right-hemisphere activity when angry faces were presented in the left hemifield (angry-neutral and angry-happy combinations). The control group showed greater right-hemisphere activity in the angry-neutral condition compared to the angry-happy condition when angry faces were present in the left hemifield.

Table 2 presents mean ABS values for each stimulus combination and for each time window of interest. Throughout the entire window of 100–3000 ms, there was a significant main effect of hemifield, *F*(1, 78) = 4.15, *p* = 0.045, $\eta_{p}^{2}=$ 0.051. Moreover, a significant three-way interaction among emotion combination, hemifield, and group was found, *F*(2, 156) = 3.09, *p* = 0.048, $\eta_{p}^{2}=$ 0.038. To unpack this interaction, *post hoc* tests were conducted across different levels of the factors (Figure 3). For the angry-happy combination, a group × hemifield comparison revealed that when angry faces appeared in the left hemifield, the stress group showed significantly higher ABS than the control group (*p* = 0.009), indicating enhanced attentional capture by left-presented angry faces under stress; by contrast, the control group showed a descriptive tendency toward a bias for happy faces (mean ABS <0).

**Table 2:** Attentional bias scores (ABS) across time windows by group and expression combination

	Angry-Neutral		Angry-Happy		Happy-Neutral	
	Left	Right	Left	Right	Left	Right
**100–3000 ms**						

Stress	0.14 ± 0.39	−0.20 ± 0.46	0.19 ± 0.46	−0.18 ± 0.47	0.12 ± 0.42	−0.12 ± 0.48
Control	0.12 ± 0.48	−0.016 ± 0.42	−0.079 ± 0.43	−0.0086 ± 0.48	0.00065 ± 0.050	−0.055 ± 0.50

**100–500 ms**						

Stress	0.085 ± 0.55	−0.014 ± 0.49	0.10 ± 0.52	−0.11 ± 0.54	−0.044 ± 0.46	−0.026 ± 0.51
Control	0.14 ± 0.65	−0.026 ± 0.56	−0.069 ± 0.67	−0.019 ± 0.45	−0.030 ± 0.64	−0.13 ± 0.69

**500–1000 ms**						

Stress	0.020 ± 0.60	−0.17 ± 0.63	0.10 ± 0.58	0.0043 ± 0.55	0.070 ± 0.69	−0.094 ± 0.059
Control	0.14 ± 0.58	−0.11 ± 0.64	0.035 ± 0.60	0.011 ± 0.67	0.041 ± 0.60	0.022 ± 0.54

**1000–2000 ms**						

Stress	0.13 ± 0.52	−0.28 ± 0.61	0.20 ± 0.58	−0.13 ± 0.60	0.18 ± 0.49	−0.17 ± 0.56
Control	0.087 ± 0.58	0.095 ± 0.54	−0.055 ± 0.53	0.11 ± 0.59	−0.085 ± 0.58	−0.054 ± 0.58

**2000–3000 ms**						

Stress	0.11 ± 0.47	−0.19 ± 0.56	0.20 ± 0.51	−0.27 ± 0.57	0.13 ± 0.060	−0.011 ± 0.52
Control	0.081 ± 0.60	−0.045 ± 0.59	−0.13 ± 0.58	−0.073 ± 0.66	0.071 ± 0.58	0.092 ± 0.48

Note: Data are presented as mean ± standard deviation (*n* = 40 participants per group). For each combination, the hemifield (left or right) indicates the side on which the higher-arousal stimulus was presented. In the angry-neutral and angry-happy combinations, this refers to the side of the angry face; in the happy-neutral combination, it refers to the side of the happy face.

Within-group comparisons further supported a leftward bias for angry stimuli in the stress group: ABS was significantly greater for left-versus-right presentation of angry faces in the angry-neutral combination (*p* = 0.008) and in the angry-happy (*p* = 0.007) combination. In the control group, when angry faces were present on the left, ABS was higher for the angry-neutral than the angry-happy combination (*p* = 0.001), suggesting that in controls, angry faces attracted more attention than neutral faces but less than happy faces in the angry-happy pairing. No main effect of group was found across the full window (*p* > 0.061).

To examine the temporal dynamics, we ran separate analyses for four interval time windows. No main effects or interactions reached significance in the 100–500 ms window (*ps* > 0.098), or in the 500–1000 ms window (*ps* > 0.054). In the 1000–2000 ms window, a significant hemifield × group interaction emerged, *F*(1, 78) = 5.59, *p* = 0.021, $\eta_{p}^{2}=$ 0.067. Follow-up tests showed that the stress group exhibited a significant left visual field (LVF) bias in this interval (*p* = 0.006). No other effects were found in this window (*ps* > 0.071). Finally, in the 2000–3000 ms window, the hemifield × group interaction remained significant, *F*(1, 78) = 4.59, *p* = 0.035, $\eta_{p}^{2}=$ 0.056, consistent with a continued LVF bias in the stress group during the last stage. No other effects reached significance in this window (*ps* > 0.072).

### ERP data of the passive viewing task

#### P1

**Figure 4:**
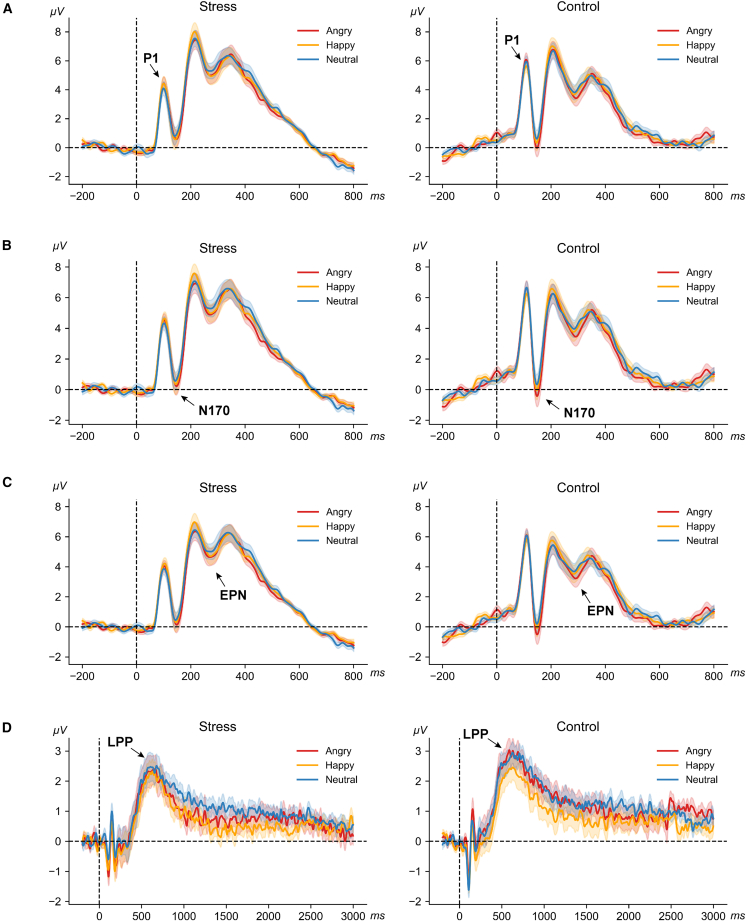
Mean ERP waveforms across emotional expressions in the passive viewing task (A) P1. (B) N170. (C) EPN. (D) LPP. Colored lines represent angry (red), happy (orange), and neutral (blue) stimuli. Shaded areas denote the standard error (SE) across participants in two groups (*n* = 40 participants per group). Dashed horizontal lines indicate zero amplitude (baseline −200 to 0 ms), and dashed vertical lines indicate stimulus onset.

For the P1 component (Figure 4A), there was a significant main effect of hemisphere, F(1, 78) = 15.43, *p* < 0.001, $\eta_{p}^{2}$ = 0.17, with higher amplitudes in the right hemisphere than in the left. There was also a significant main effect of group, F(1, 78) = 5.13, *p* = 0.026, $\eta_{p}^{2}$ = 0.062, with participants in the control group exhibiting larger P1 amplitudes than those in the stress group. No interaction effects were found (*ps* > 0.28).

#### N170

For the N170 component (Figure 4B), there was a significant main effect of expression, F(2, 156) = 4.38, *p* = 0.014, $\eta_{p}^{2}$ = 0.053, with angry expressions eliciting larger N170 amplitudes than happy (*p* = 0.023) and neutral (*p* = 0.033) expressions. There was also a significant main effect of hemisphere, F(1, 78) = 21.81, *p* < 0.001, $\eta_{p}^{2}$ = 0.22, with larger amplitudes in the right hemisphere than in the left. No group or interaction effect was found (*ps* > 0.39).

However, inspection showed that high P1 amplitudes affected the absolute N170 values. To address this, peak-to-peak P1-N170 amplitudes were calculated.^79^ This analysis showed that the right hemisphere N170 was significantly larger than the left (F(1, 78) = 14.16, *p* < 0.001, $\eta_{p}^{2}$ = 0.15). The main effect of emotion remained unchanged. Additionally, a significant group difference emerged: The control group had larger N170 amplitudes than the stress group (F(1, 78) = 7.58, *p* = 0.007, $\eta_{p}^{2}$ = 0.089). No interaction effects were observed (*ps* > 0.16).

#### EPN

The EPN results (Figure 4C) showed a significant main effect of expression, F(2, 156) = 3.82, *p* = 0.024, $\eta_{p}^{2}$ = 0.047, with angry faces eliciting significantly larger EPN amplitudes than happy (*p* = 0.030) and neutral (*p* = 0.017) faces. Besides, there was also a significant main effect of hemisphere, F(1, 78) = 87.10, *p* < 0.001, $\eta_{p}^{2}$ = 0.53, with larger amplitudes in the right hemisphere than the left. The group difference did not reach significance (*p* = 0.084), and no interactions were found (*ps* > 0.36).

#### LPP

For the LPP (Figure 4D), a significant main effect of group was found, F(1, 78) = 4.99, *p* = 0.028, $\eta_{p}^{2}$ = 0.060, showing the control group had significantly larger LPP amplitudes than the stress group. No other main effects or interactions were significant (*ps* > 0.51).

Topographical maps for each ERP component are provided in Figure S1.

#### Event-related spectral perturbation in the passive viewing task

**Figure 5:**
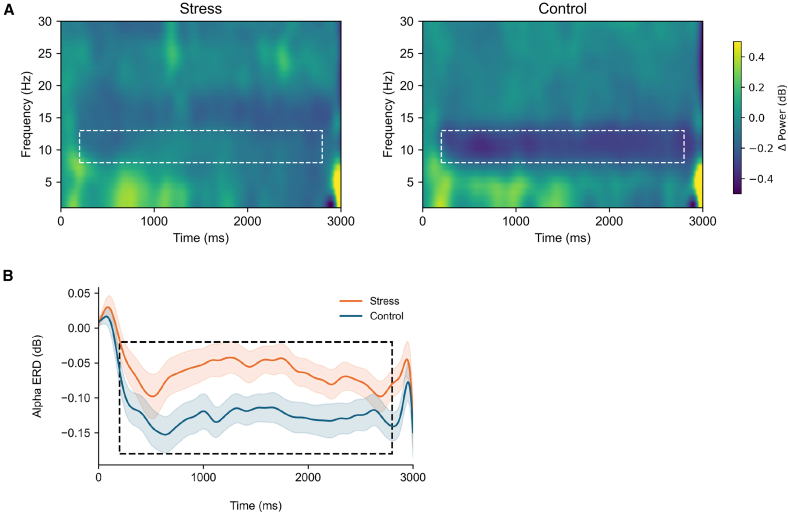
Frontal alpha-band responses in the passive viewing task (A) Time-frequency representations aligned to stimulus onset. The white dashed box indicates the alpha-band within the analysis window, showing reduced alpha event-related desynchronization (ERD) in the stress group (*n* = 40 participants per group). (B) Time-resolved alpha ERD. Shaded areas denote the standard error (SE) across participants in two groups (*n* = 40 participants per group), and the black dashed box indicates the analysis window.

For the alpha event-related spectral perturbation (alpha ERD) (Figure 5), a significant main effect of group was found (F(1, 76) = 5.73, *p* = 0.019, $\eta_{p}^{2}$ = 0.070), indicating that the stress group showed less alpha ERD than the control group. No other significant effects were observed (*ps* > 0.29). Additional analyses from alternative frontal electrode clusters are reported in the supplemental materials Table S1.

## Discussion

Acute stress is known to modulate attention and emotional reactivity.^29^^,^^39^ Here, we examined how it influences attentional and emotional processing in socially anxious individuals (Table 3). Following TSST induction, participants completed two tasks: an ATB task and a passive viewing task with behavioral, physiological, and neural recordings. In the ATB task, the stress group showed an LVF bias during late-stage attentional processing, and in the passive viewing task, they exhibited overall reduced ERP amplitudes. Intriguingly, although acute stress significantly disrupted attentional allocation and selective processing, it did not enhance early-stage attentional bias to threat.

**Table 3:** Demographic characteristics of participants

	Stress (M ± SD)	Control (M ± SD)	*t*	*p*	*95% CI*
n	40	40	–	–	–
Age	20.50 ± 2.15	20.43 ± 2.10	0.16	0.88	[-0.87, 1.02]
LSAS	55.25 ± 20. 20	55.23 ± 15.71	0.006	0.99	[-8.03, 8.08]
DASS-21	39.03 ± 12.14	39.83 ± 11.79	−0.30	0.77	[-6.13, 4.53]
IUS	71.63 ± 15.41	72.25 ± 14.87	−0.19	0.85	[-7.37, 6.12]
BES-pre	58.30 ± 5.75	58.10 ± 5.278	0.16	0.87	[-2.26, 2.66]

Note: n = number of participants; M = mean; SD = standard deviation; CI = confidence interval; LSAS = Liebowitz Social Anxiety Scale; DASS-21 = Depression, Anxiety, Stress Scale-21 items; IUS = Intolerance of Uncertainty Scale; BES = Basic Empathy Scale. Group differences were assessed using two-tailed independent-samples t-tests.

To ensure stress manipulation, we verified the effectiveness of stress induction through subjective and physiological markers. As expected, significantly elevated self-reported stress levels in the stress group confirmed successful subjective stress induction. However, we did not observe group differences in salivary cortisol or sAA levels, providing limited evidence for HPA axis activation in the present study. Several factors may account for it. First, the use of a VR-based stress paradigm may have reduced the intensity of the psychosocial stressor compared to traditional in-person TSST procedures. While VR paradigms can reliably elicit subjective stress, they do not always produce consistent hormonal changes.^75^^,^^80^^,^^81^ Indeed, several studies employing the Open TSST-VR have similarly reported increased subjective stress, but failed to observe elevations in cortisol and sAA.^73^^,^^82^^,^^83^

Second, characteristics of the present sample may have contributed to the blunted endocrine effects. Previous research has shown that men often have greater cortisol reactivity than women.^84^ Given that the majority of our sample were female participants, this sex distribution may have caused relatively low cortisol responses. Moreover, socially anxious individuals have been shown to exhibit altered glucocorticoid regulation of the HPA axis^85^ or heightened context-dependent stress reactivity, which may lead to greater variability or attenuation of cortisol.^86^ Such a pattern may reflect an adaptive coping strategy for repeated or chronic stress. Meanwhile, the rise in sAA across both groups suggests a general sympathetic activation, possibly due to the inherently evaluative and socially demanding nature of social tasks for socially anxious participants.

### Acute stress biases sustained visuospatial allocation toward the left visual field

Visual attention shifts dynamically based on arousal, cognitive effort, and resource availability.^87^ In the ATB task, acute stress did not enhance attentional bias toward angry faces as initially predicted. Instead, the stress group exhibited a sustained LVF preference during the later stimulus window (1000–3000 ms), whereas both groups showed comparable early engagement (100–1000 ms). This temporal dissociation suggests that stress primarily affects the maintenance phase of spatial attention, rather than initial orienting, promoting a right-lateralized processing mode.

This leftward bias can be understood in light of right-hemisphere dominance for visuospatial and emotional processing.^88^^,^^89^^,^^90^ Neuroimaging studies have also reported stress-related modulation of hemispheric asymmetry.^91^^,^^92^ In this context, our findings suggest that acute stress reinforces sustained spatial weighting toward right-hemispheric streams, rather than dynamically redistributing attention across competing affective input.

Two features of our data support this stress-specific outcome. First, the LVF bias emerged only during the late time window and was present exclusively in the stress group. Second, it was independent of stimulus pairing or physical salience, indicating that the effect reflects altered allocation dynamics instead of perceptual asymmetries. By contrast, the control group gradually shifted attention over time (favoring happy faces), consistent with the vigilance-avoidance pattern often seen in SAD.^93^ This shift was accompanied by larger SCRs in happy-neutral than in angry-neutral face pairs. Importantly, the LVF bias was most pronounced when angry faces were present, suggesting that emotional threat further amplifies the sustained spatial pattern under stress.

These results challenge the functional equivalence view, which posits flexible hemispheric redistribution under stress^94^^,^^95^ and instead suggest reduced attentional switching. Acute stress appears to enhance sustained lateralized allocation during later processing states, and potentially reflects a shift toward automatic, energetically efficient processing routes.^96^^,^^97^ This interpretation aligns with evidence that the brain favors overlearned pathways under stress, at the cost of flexible cognitive adjustments.^29^^,^^98^^,^^99^ Functionally, such a shift supports rapid threat detection but may be maladaptive when nuanced social evaluation or priority switching is required. Although Zheng et al. reported difficult disengagement from angry faces in a high social anxiety sample using a similar task,^100^ the inconsistency in our study may relate to differences in social anxiety severity or cultural interpretation of facial expressions. Moreover, our central fixation and passive view design reduced opportunities for exploratory, emotion-guided shifts compared to active tasks.

### Acute stress depletes attentional resources and reduces attentional control

In the passive viewing task, acute stress was associated with widespread reductions in ERP amplitudes, spanning from early sensory processing (P1), face structural encoding (peak-to-peak N170), and sustained attentional engagement (LPP). This generalized suppression extends beyond the spatial inflexibility in the ATB task and suggests a broader decreased regulation of cortical responsiveness and depletion of available attentional resources under stress.

Complementing the ERP findings, frontopolar alpha ERD was significantly smaller in the stress group. Alpha ERD at frontal sites is commonly considered to reflect the engagement of frontal control-related processes under cognitive demand.^101^^,^^102^ Its reduction may therefore indicate attenuated top-down attentional regulation. This interpretation aligns with prior evidence that anterior prefrontal regions are particularly sensitive to stress-related influences,^103^^,^^104^ and that inefficient frontal pole engagement is associated with limited emotional control in socially anxious individuals.^105^

Together, converging ERPs and alpha ERD findings suggest that acute stress compromises attentional resources required for perceptual filtering and executive regulation, resulting in a generalized reduction in attentional control capacity.^106^^,^^107^^,^^108^ Although the passive viewing task imposed minimal top-down demands, the reduced alpha ERD in the stress group may still signal diminished readiness for regulatory engagement. Such depletion may be especially consequential for socially anxious individuals, who are hypersensitive to social stress and may struggle to sustain goal-directed attention in socially salient contexts.

### Acute stress preserves early automaticity but impairs late-stage processing

Contrary to our hypothesis, acute stress did not elevate vigilance or neural sensitivity to angry expressions in the passive viewing task. Instead, our findings suggest that while early-stage emotional responses remain largely preserved, sustained processing is compromised under stress. Previous work has found larger P1 amplitudes to emotional expressions—particularly angry, happy, or fearful faces—compared to neutral faces or non-face objects.^109^^,^^110^^,^^111^ The absence of P1 emotional modulation here instead supports the view that P1 is primarily driven by low-level visual features rather than higher-order face perception,^112^ which explains why it remained unaffected.

Unlike P1, we replicated the emotional modulations in the N170 and EPN components, in line with prior research.^113^ Stronger N170 responses to angry relative to happy and neutral faces highlight its sensitivity to high-arousal, especially threat-related, social cues.^114^ A similar pattern emerged in the EPN, which also showed heightened sensitivity to angry expressions, suggesting automatic attentional capture by threat-related stimuli even under limited attentional resources. The main hemisphere effects observed in the P1, N170, and EPN components further support right-hemisphere specialization for emotional processing.

In contrast to the robust early perceptual encoding, late-stage emotional engagement appeared attenuated. The LPP, which reflects prolonged threat processing,^11^^,^^115^ was not heightened in our task. At the descriptive level, the control group exhibited elevated LPP responses to angry faces, whereas the stress group showed no differentiation across expressions. The low affective salience of the stimuli and the passive task design may have limited deeper emotional engagement, as weak emotional cues often fail to elicit the amygdala-mediated modulation of the visual cortex, especially under cognitive strain.^116^^,^^117^

Overall, the current findings demonstrate a dynamic model of attention-emotion interaction. Emotional processing starts with independent and automatic early stages in the visual cortex and progresses to later stages that are highly dependent on available attentional capacity.^118^^,^^119^^,^^120^ When that capacity is consumed by stress, high-order emotional engagement falters. Accordingly, emotional differentiation by valence emerges only when attentional resources allow focused and prioritized processing, as amygdala reactivity in emotion processing also requires top-down attention.^121^ Therefore, the availability of attentional resources shapes not only how but whether emotions are fully processed and reach conscious representation under stress.

### Limitations of the study

Several limitations should be acknowledged. First, cortisol responses did not show group differences in the present sample, which limits firm conclusions about HPA axis activation in response to the stress. Future studies may benefit from using more intensive or ecologically immersive stress paradigms and including additional physiological markers to better capture endocrine responses. Second, the sample was not fully representative: Only right-handed participants were included, and the majority were female. Although restricting handedness is common in EEG research to reduce variability in hemispheric lateralization, these sampling characteristics limit the generalizability of the observed laterality and stress effects. Future studies should recruit more diverse and balanced samples to determine whether the present findings are replicated across different populations.

Third, the absence of a non-anxious control group precludes direct comparisons of attentional and emotional processing across populations, making it difficult to determine the specificity of the observed stress effects to socially anxious individuals. Future work should include appropriate control groups and larger samples to examine stress-by-trait interactions. Finally, although the hypothesized threat-specific amplification was not observed, stress may influence emotional processing indirectly through attentional resource depletion. Future studies should therefore employ designs that more directly disentangle the dynamic interplay between stress, attention, and emotion.

## Resource availability

### Lead contact

Requests for further information and resources should be directed to and will be fulfilled by the lead contact, Mozhao Li (m.z.h.li@essb.eur.nl).

### Materials availability

This study did not generate new materials.

### Data and code availability

• All data reported in this article have been deposited at the Open Science Framework project repository (https://doi.org/10.17605/OSF.IO/RMSZQ) and are publicly available as of the date of publication.
• This paper does not report original code.
• Any additional information required to reanalyze the data reported in this paper is available from the lead contact upon request.

## Acknowledgments

This work was supported by 10.13039/501100004543China Scholarship Council (202208060157). We also thank all the participants of this study. Graphical abstract is created with BioRender.com.

## Author contributions

Conceptualization, M.L., B.v.D., G.D., and M.J.W.; methodology, M.L., B.v.D., G.D., and M.J.W.; investigation, M.L.; formal analysis, M.L.; data curation, M.L.; writing – original draft, M.L.; writing – review and editing, A.H.K.W. and M.J.W.; supervision, A.H.K.W. and M.J.W.

## Declaration of interests

The authors declare no conflict of interests.

## STAR★Methods

### Key resources table

**Table:** 

REAGENT or RESOURCE	SOURCE	IDENTIFIER
**Deposited data**		

Behavioral and physiological data; analysis code	This paper; Open Science Framework	https://doi.org/10.17605/OSF.IO/RMSZQ

**Software and algorithms**		

E-Prime 3.0	Psychology Software Tools	https://pstnet.com/products/e-prime/
MNE-Python 1.11.0	Gramfort et al.^122^	https://mne.tools/stable/index.html
NeuroKit2	Makowski et al.^123^	https://pypi.org/project/neurokit2/
Python 3.11.5	Python Software Foundation	https://www.python.org/downloads/release/python-3115/
SPSS 29.0.1	IBM	https://www.ibm.com/support/pages/downloading-ibm-spss-statistics-2901
Adobe Photoshop 2023	Adobe Inc.	https://www.adobe.com/products/photo.html
G∗Power 3.1.9.6	Faul et al.^124^	https://www.psychologie.hhu.de/arbeitsgruppen/allgemeine-psychologie-und-arbeitspsychologie/gpower

### Experimental model and study participant details

Ninety Dutch-speaking undergraduate psychology students from Erasmus University Rotterdam participated in exchange for course credits. The study employed a between-subject design with two groups: a stress group and a control group. Potential participants were initially screened online (*n* = 162), and individuals scoring ≥ 30 on the Liebowitz Social Anxiety Scale (LSAS)^125^ were selected to identify socially anxious individuals. No formal diagnostic interview was conducted. This cut-off has been shown to provide a balance between specificity and sensitivity for distinguishing SAD and non-anxious individuals.^126^

All participants had normal or corrected-to-normal vision. Exclusion criteria included the use of psychoactive medication or oral contraceptive within the past month, a history of cardiac or neurological diseases, and habitual smoking (≥ 5 cigarettes per day) due to potential hormonal effects.^127^ Before the experimental session, participants completed the Depression Anxiety Stress Scale (DASS-21),^128^ Intolerance of Uncertainty Scale (IUS),^129^ and Basic Empathy Scale (BES).^130^ Of the nighty participants enrolled in the study, ten were excluded based on predefined criteria and data quality. The final sample consisted of eighty participants (*n* = 40 per group; 35 females in each group). No group differences were observed in questionnaire measures (all *ps* > 0.49; see Table 3). The study was approved by the local ethics committee (ETH2223-0795) and conducted in accordance with the 1964 Declaration of Helsinki. All participants provided written informed consent.

### Method details

#### Stimuli

Task 1 - attentional threat bias (ATB) task: Stimuli were selected from the Karolinska Database of Emotional Faces (KDEF),^131^ including angry, neutral and happy expressions from 12 female and 12 male models. Images were presented as gender-matched emotional pairs (angry-neutral, angry-happy, happy-neutral), flickering at 12 or 15 Hz, and subtending a viewing angle of 14°. Emotional pairing, flicker frequency, and visual hemifield were counterbalanced across trials.

Task 2 - passive viewing task: Stimuli were selected from the Radboud Face Database (RaDF),^132^ including angry, neutral, and happy expressions from 16 female and 16 male models. Each image was centrally presented with a viewing angle of 7.2°.

All images were converted to grayscale and edited in Adobe Photoshop 2023 to remove prominent hair and mustache features. Both EEG tasks were designed in E-Prime 3.0 (Psychology Software Tools Inc., USA) and presented on a monitor with refresh rate of 120 Hz.

#### Subjective measures

Momentary stress and mood were rated through a visual analog scale (VAS) consisting of seven items: stress, relaxation, physical discomfort, perceived controllability, desire to leave, need for support, and overall emotional state, each rated from 0 to 1.^133^ In the passive viewing task, participants rated the valence and arousal of each image using affective sliders ranging from 0 to 1.^134^ Valence ranged from unpleasant to pleasant; arousal ranged from calm to wide awake.

#### Procedure

Participants first completed the questionnaires online before the lab visit. They were randomly assigned to the stress or control group, matched for demographics (see Table 3). All sessions ran between 12:30 and 5:30 p.m. to minimize circadian cortisol variability.

Six saliva samples were collected using Salivettes (Sarstedt, Nümbrecht, Germany), each accompanied by a VAS rating (Figure 1). The first sample was taken upon arrival, followed by EEG and VR headset setup (HTC VIVE Pro Eye; HTC Corporation, Taipei, Taiwan). The second sample was collected 25 min later and served as a baseline to stabilize potential emotional fluctuations.

Participants then completed the TSST/placebo-TSST in VR.^73^ The stress group underwent the TSST-VR, which consisted of a 5-min preparation, a 5-min job interview speech delivered to a virtual panel, and a 5-min mental arithmetic task of serial subtraction from 2043 in steps of 17. The control group completed a non-evaluative placebo-TSST involving a neutral speech and easy arithmetic task (addition by 15). A third saliva sample and VAS ratings were collected immediately after to assess stress induction efficacy.

EEG recording followed in a separate room. Participants were seated 1 m from the monitor and fitted with electrooculogram (EOG), electrocardiogram (ECG) and galvanic skin response (GSR) electrodes. The ATB task included two blocks of 144 image combinations in randomized order while participants maintained fixation on a central cross. Each trial lasted 3000 ms with an inter-trial interval of 4500–5500 ms. During intervals, the black fixation cross changed to light gray 3–5 times, and participants reported these changes to ensure sustained fixation. The fourth and fifth saliva samples were taken after each block. In the passive viewing task, participants viewed 96 images for 3000 ms each, followed by valence and arousal ratings. The final saliva sample and post-experiment BES were collected at the end of the session.

#### Physiological data acquisition

EEG was recorded using a 64-channel BioSemi Active Two system (BioSemi B.V., Amsterdam, the Netherlands) with electrodes positioned according to the international 10–20 system. The system’s Common Mode Sense/Driven Right Leg (CMS/DRL) electrodes were applied to reduce common-mode noise and enhance signal quality. Data were sampled at 512 Hz, and impedance was maintained below 10 kΩ. Vertical and horizontal EOGs were recorded from four electrodes (above/below the left eye; outer canthi of both eyes), ECG from an electrode on the left lower rib, and GSR from the index and middle fingers of the left hand.

#### Physiological measure processing

##### Saliva analysis

Samples were stored at −20° until analysis. Cortisol concentrations were measured using Dissociation-Enhanced Lanthanide Fluorescent Immunoassay (DELFIA),^135^ and salivary alpha amylase (sAA) via CNP-G3 colorimetric assay at the University of Trier, Germany. For cortisol, the intra- and inter-assay coefficients of variation were 4.0–6.7% and 7.1–9.0%, respectively. For sAA, they were 2.8–6.3% and 5.5–7.6%, respectively. Raw concentrations and area under the curve (AUC) values were calculated to capture acute and cumulative stress responses.^136^ AUC with respect to ground (AUCg) was calculated from six timepoints; AUC with respect to increment (AUCi) was calculated from the second to the sixth timepoint.

##### EEG preprocessing

EEG data preprocessing was performed using the MNE-Python 1.11.0.^137^ Raw data were bandpass filtered between 0.01 and 40 Hz and notch filtered at 50 Hz to remove line noise. The data were then visually inspected to identify disconnected or noisy electrodes, which were interpolated from neighboring channels to preserve spatial information. Trials contaminated by large head movements or faulty electrodes were discarded.

Independent component analysis (ICA) was applied to the continuous EEG date to decompose the signal into 64 independent components. Components reflecting eye blinks, eye movements, cardiac activity, or muscle artifacts were identified based on ICLabel classification^122^ and detailed visual inspection of topographies, power spectra, and time courses, and were subsequently removed. The cleaned data were then re-referenced to the average of all electrodes. Epochs were extracted from −200 ms to 3000 ms relative to stimulus onset, and segments exceeding 150 μV were excluded. Baseline correction was applied by removing the mean value of the 200 ms pre-stimulus interval.

In the ATB task, an average of 0.16 electrodes were interpolated per participant. The mean numbers of valid trials in the stress group were: angry-neutral: 43.05, angry-happy: 43.2, happy-neutral: 43.3; and in the control group: 41.25, 41.93, and 41.33, respectively. In the passive viewing task, an average of 0.18 electrodes were interpolated per participant. The mean numbers of valid trials in the stress group were: angry: 30.5, happy: 30.8, neutral: 30.7; and in the control group: 31.18, 31.43, and 30.93, respectively. No significant differences were found between groups or across conditions within groups (*ps* > 0.11).

##### ECG processing

ECG and GSR data were preprocessed using NeuroKit2 0.2.13,^138^ bandpass filtered from 0.5 to 40 Hz, and baseline corrected at −1000 ms. HR was derived from RR intervals. In the ATB task, HR were analyzed across 0–1000 ms (deceleration), 1000–3000 (acceleration), 3000–7500 ms (post-stimuli recovery), and 0–7500 ms (overall). In the passive viewing task, where HR deceleration was primarily observed, HR were analyzed across 0–3000 ms.

##### GSR processing

GSR data were bandpass filtered from 0.01 to 5 Hz and analyzed using the peak-to-valley method. In both tasks, valleys were defined as the minimum value within 1000–4000 ms after stimulus onset, and peaks as the maximum within 1500–7500 ms. Only peaks following valid valleys within a trial were retained, and responses below 0.02 μS were excluded.^123^ Specifically, in the passive viewing task, trials shorter than 7500 ms were excluded (mean exclusion rate: 10.13%) to prevent overlap of response windows across trials. Participants were included in SCR analyses only if they had at least five valid trials per condition (ATB task: angry-happy, angry-neutral, happy-neutral; passive viewing task: angry, happy, and neutral) to ensure stable condition-level estimates.

##### ssVEP processing and attentional bias scores in the ATB task

Spectral analyses were conducted on the preprocessed EEG data. Fast Fourier Transform was first applied to extract spectral power at 12 Hz and 15 Hz. The resulting power distributions were visualized as scalp topographies to identify the most responsive regions. Seven electrodes (O1, O2, Oz, PO7, PO8, P9, P10) over parieto-occipital and occipital areas were selected for subsequent analyses. Time-frequency decomposition was then performed using Morlet continuous wavelets across 1–40 Hz in 10 logarithmically spaced steps. The number of wavelet cycles was set as frequency/2 (e.g., 6 cycles for 12 Hz and 10 for 20 Hz). Amplitudes were averaged across the selected channels, expression types within each face combination, and hemifields, and were then summed across frequencies to obtain responsive amplitudes for each expression type in each hemifield.

For time-domain analyses, mean amplitudes were extracted from four windows: 100–500 ms, 500–1000 ms, 1000–2000 ms, and 2000–3000 ms, as well as the global 100–3000 ms window.^14^ Attentional bias scores (ABS) were calculated as follows: angry-neutral: (angry - neutral)/(angry + neutral); angry-happy: (angry - happy)/(angry + happy); happy-neutral: (happy - neutral)/(happy + neutral). Higher positive ABS values indicate a greater attentional preference toward the more arousing stimulus in each pair.

##### EEG processing in the passive viewing task

ERPs were analyzed for four components: P1, N170, EPN, and LPP. Responsive electrode clusters selected based on topographic inspection and prior literature.^110^^,^^139^^,^^140^^,^^141^ The P1 was measured at O1/O2/PO7/PO8/P9/P10 cluster between 80 and 120 ms; the N170 at PO7/PO8/P9/P10 cluster between 130 and 200 ms; the EPN at P7/P8/PO7/PO8/P9/P10 cluster between 240 and 300 ms; and the LPP at the midline Cz/CPz/Pz/Poz cluster between 400 and 1000 ms. Components amplitudes were calculated as the mean voltage within the defined windows.

For alpha power analysis, two participants (one from the stress and one from the control group) were excluded due to poor signal quality. Alpha power was extracted in the 8–13 Hz frequency range from frontal electrodes (Fp1, Fp2, Fpz) and log-transformed. The baseline period was defined as −200 to 0 ms relative to stimulus onset, and the stimulus period was defined as 200 to 2800 ms post-stimulus. Negative values reflect a decrease in alpha power relative to baseline (desynchronization, ERD), whereas positive values an indicate increase (synchronization, ERS).

### Quantification and statistical analysis

A prior power analysis was conducted using G∗Power 3.1.9.7^124^ (effect size f = 0.25, α = 0.05, power = 0.95), indicating a minimum required sample size of 84 participants, based on the expected interaction between stress induction (group) and facial expression. Ten participants were excluded due to later-reported left-handedness (*n* = 3), medication use (*n* = 3), technical recording issues (*n* = 2), and delayed task onset outside the required timeline (*n* = 2), resulting in a final sample of 80 participants, with 40 in the stress group (35 females) and 40 in the control group (35 females). Statistical analyses were performed using Python 3.11.5 and SPSS 29.0.1 (IBM, USA). The Greenhouse-Geisser correction was applied when the sphericity assumption was violated. Bonferroni corrections were applied for all analyses. All *p* values of <0.05 were considered significantly different. Exact sample sizes for each analysis are reported in the corresponding data processing procedures and figure legends.

#### Behavioral data

Demographic characteristics were compared between groups using two-tailed independent t-tests. Stress level scores from the VAS (question: How stressed do you feel at the moment?) were analyzed with a two-way repeated measures analysis of variance (rmANOVA) (group × timepoint). Affective ratings were analyzed using a two-way rmANOVA (group × expression).

#### EEG data

In the ATB task, ABS values were analyzed with a three-way rmANOVA (group × expression × hemifield) across each time window. In the passive viewing task, P1, N170, and EPN amplitudes were analyzed using three-way rmANOVA (group × expression × hemisphere). LPP amplitudes were analyzed using a two-way rmANOVA (group × emotion expression).

#### Physiological data

Cortisol and sAA levels raw concentrations were analyzed using a two-way rmANOVA (group × timepoint) (t-30, t-pre, t+00, t+15, t+30, t+50). AUCg and AUCi were compared between groups using independent t-tests. GSR and HR data in both tasks were analyzed with two-way rmANOVA (group × expression).
