# Supplementary material for: Acute stress impairs attentional control and emotional processing in social anxiety
Source: iScience. 2026 Apr 17;29(5):115767. doi: 10.1016/j.isci.2026.115767 (PMC13157114; doi:10.1016/j.isci.2026.115767)
Supplement: Document S1. Figure S1 and Table S1 [file mmc1.pdf]

iScience, Volume *29*

Supplemental information

Acute stress impairs attentional control

and emotional processing in social anxiety

Mozhao Li (李墨钊), Alex H.K. Wong, Bernadette von Dawans, Gregor Domes, and Matthias J. Wieser

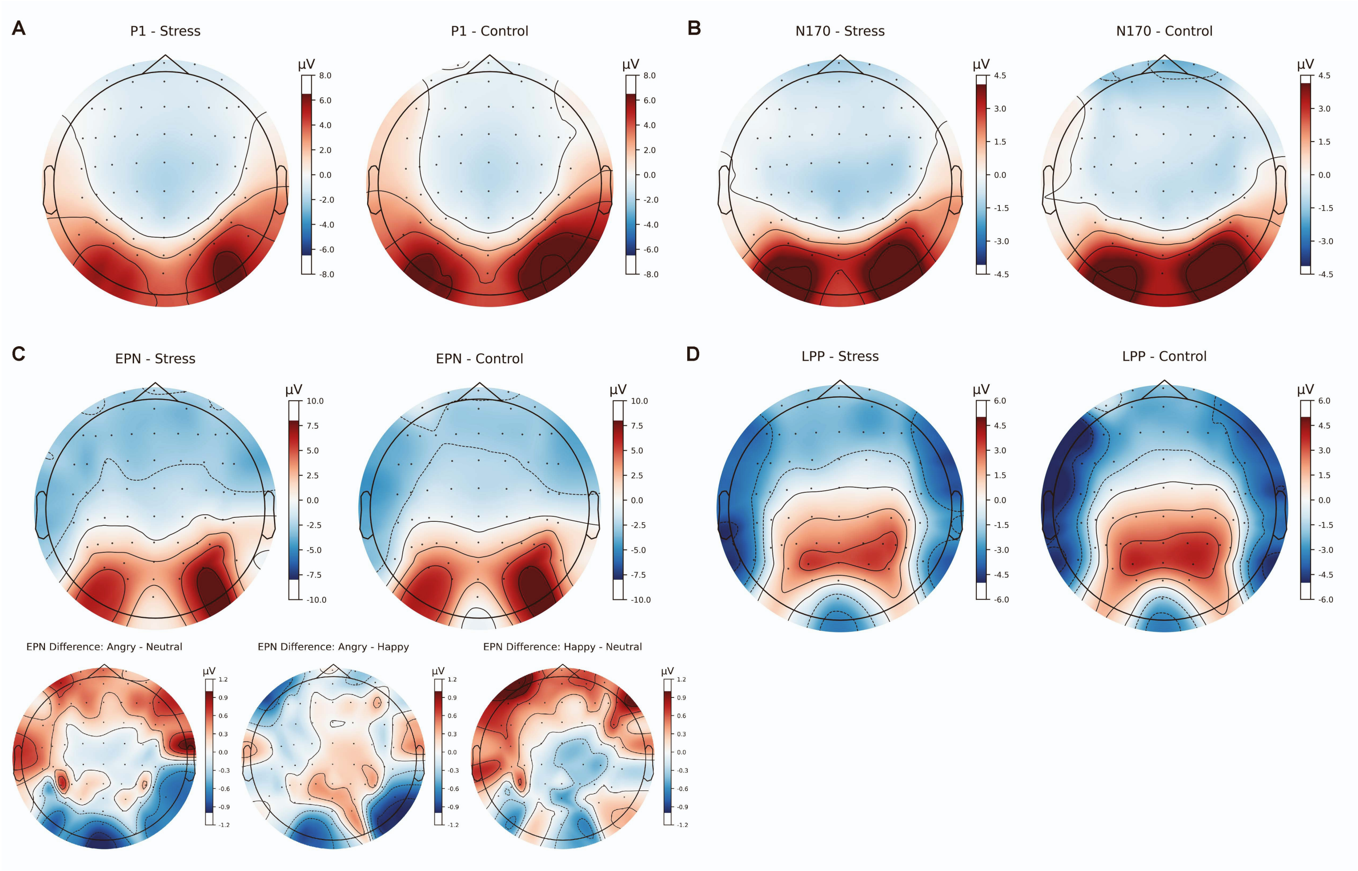

Figure S1 Scalp topographies of ERP components in the stress and control groups, related to Figure 4. (A) P1 (80-120 ms). (B) N170 (130-200 ms). (C) EPN (240-300 ms). (D) LPP (400-1000 ms).

Table S1 Alpha-ERD across electrode clusters

Cluster	Stress (M ± SD)	Control (M ± SD)	F	P	η_p^2
F3/F4	-0.77 ± 1.33	-1.03 ±1.33	0.76	0.39	0.10
F3/F4/F7/F8	-0.83 ± 1.43	-1.17 ±1.32	1.19	0.28	0.015
F3/F4/F5/F6/F7/F8	-0.84 ± 1.43	-1.13 ± 1.26	0.90	0.35	0.011
F3/F4/F7/F8/Fpz/Fz	-0.79 ± 1.41	-1.20 ± 1.32	1.81	0.18	0.023

[a]*Alpha-ERD results for electrode clusters : F3/F4; F3/F4/F7/F8; F3/F4/F5/F6/F7/F8; F3/F4/F7/F8/Fz.*
